# Detection of Platelet-Activating Antibodies Associated with Vaccine-Induced Thrombotic Thrombocytopenia by Flow Cytometry: An Italian Experience

**DOI:** 10.3390/v14061133

**Published:** 2022-05-24

**Authors:** Francesca Cesari, Silvia Sorrentino, Anna Maria Gori, Angela Rogolino, Raimondo De Cristofaro, Betti Giusti, Elena Sticchi, Erica De Candia, Rossella Marcucci

**Affiliations:** 1 Atherothrombotic Disease Unit, Department of Experimental and Clinical Medicine, University of Florence, Azienda Ospedaliera Universitaria Careggi, 50141 Florence, Italy; cesarif@aou-careggi.toscana.it (F.C.); annamaria.gori@unifi.it (A.M.G.); rogolinoa@aou-careggi.toscana.it (A.R.); betti.giusti@unifi.it (B.G.); elena.sticchi@unifi.it (E.S.); rossella.marcucci@unifi.it (R.M.); 2 Malattie Emorragiche e Trombotiche, Fondazione Policlinico Universitario Agostino Gemelli IRCSS, 00168 Rome, Italy; silvia.sorrentino@unicatt.it (S.S.); raimondo.decristofaro@unicatt.it (R.D.C.); 3 Dipartimento di Medicina e Chirurgia Traslazionale, Università Cattolica del Sacro Cuore, 00168 Rome, Italy

**Keywords:** vaccine-induced thrombocytopenia and thrombosis (VITT), diagnosis, flow cytometry, HIPA, heparin-induced thrombocytopenia and thrombosis (HIT)

## Abstract

Rare cases of thrombocytopenia and thrombosis after anti-COVID-19 adenovirus-associated mRNA vaccines (VITT) due to platelet-activating anti-platelet-factor 4 (PF4)/polyanion antibodies have been reported. VITT laboratory diagnosis, similarly to heparin-induced thrombocytopenia (HIT) diagnosis, requires immunoassays for anti-PF4/polyanion antibodies identification, such as ELISA assays and platelet-activating functional tests, such as heparin-induced platelet activation test (HIPA), to confirm their pathogenicity. We compared the flow cytometry (FC) measurement of platelet p-selectin exposure to the gold standard functional test HIPA for diagnosis confirmation in 13 patients with a clinical VITT syndrome (6M/7F; median age 56 (33–78)) who resulted positive to anti-PF4/polyanion antibodies ELISA assays (12/13). FC and HIPA similarly identified three different patterns: (1) a typical non-heparin-dependent VITT pattern (seven and six patients by FC and HIPA, respectively); (2) low/no platelet activation in patients under IvIg therapy (five out of five and two out of four patients by FC and HIPA, respectively); (3) a HIT pattern. Antibodies investigated by FC became negative after 7, 17, and 24 days of therapy in three patients. FC measurement of P-selectin exposure was as sensitive as HIPA but simpler to detect anti-PF4/polyanion antibodies in VITT patients. FC could reliably discriminate VITT from HIT, thus helping for the choice of the anticoagulant.

## 1. Introduction

The current pandemic of coronavirus disease 2019 (COVID-19) owed to the severe acute respiratory syndrome coronavirus 2 (SARS-CoV-2) has determined a critical public health threat with millions of infected worldwide [1]. Presently, vaccination represents the most effective weapon in the fight against SARS-CoV-2, reducing the number of severe cases or deaths in countries where vaccines are provided. Four different vaccines to prevent symptomatic COVID-19 have been approved by the European Medical Agency: BNT162b2 (Pfizer-BioNTech), mRNA-1273 (Moderna), Vaxzevria (AstraZeneca), and Ad26.COV2.S (Johnson & Johnson) [2]. Notably, the latter two are adenoviral vectors encoding SARS-CoV-2 spike glycoprotein [3].

Unfortunately, several cases of thrombotic events in combination with thrombocytopenia and bleeding have been reported after adenovirus-associated mRNA vaccination [4,5,6,7]. The clinical picture was similar to symptoms and signs that develop in association with heparin-induced thrombocytopenia and thrombosis (HIT). Thrombotic events were both in arterial and venous sites. Venous thrombosis occurred mostly in unusual sites, such as cerebral veins and subdural venous sinus and splanchnic veins. In many patients, deep vein thrombosis and pulmonary embolism simultaneously occurred. Thrombotic events were more severe than in HIT, and the mortality rate was close to 50% in initial reports [4,5,6] and 22% in a larger cohort more recently reported [7]. The new nosographic entity named vaccine-induced immune thrombotic thrombocytopenia (VITT) was mainly observed in women under 55 years of age, between 4 and 16 days after Vaxzevria shot and, in a few more cases, after Ad26.COV2.S administration [4,5,6,7].

In VITT patients, the detection of anti-platelet factor 4 (PF4) IgG antibodies-activating platelets, unrelated to the use of heparin, was reported [6,7,8,9], showing similarities with autoimmune HIT (aHIT) [10]. PF4/heparin enzyme immunoassays (EIA) are widely available to diagnose HIT, and since the occurrence of VITT, they have been used for the diagnosis of the disease. Several commercially available methods, such as chemiluminescence or ELISA, recognize anti-PF4/heparin antibodies. These assays generally have high sensitivity (80–100%) for HIT antibodies but suffer from low specificity due to the detection of antibodies that are not platelet-activating and do not elicit HIT (false positive). HIT immunoassays, however, may not be specific for the detection of VITT-related antibodies. In fact, it has already been reported that a specific chemiluminescence HIT immunoassay (HemosIL Acustar HIT IgG assay) was not able to identify VITT-related antibodies [11]. In addition, similarly to HIT antibodies, not all anti-PF4/polyanion antibodies identified by EIAs are pathogenic. A previous study showed that, among 492 health care workers vaccinated with Vaxzevria, anti-PF4/polyanion antibodies without platelet-activating properties were found after vaccination in six individuals with normal platelet counts and no thrombosis [12]. Therefore, a functional test for the identification of pathogenic platelet-activating antibodies, as for HIT diagnosis, is required to confirm the diagnosis and to identify clinically relevant antibodies. For HIT diagnosis, the gold standard functional test is the heparin-induced platelet-activation (HIPA) assay. This assay tests the ability of patients’ plasma/serum to aggregate control platelets in the presence of low (0.3 IU/mL) and high (100 IU/mL) heparin concentrations. The test takes a long time (6–7 h), as it requires platelet washing procedures and expert operators and is completely manual. For these reasons, it is not available in most laboratories, and few highly specialized laboratories are equipped to perform it [13].

The flow cytometry (FC) assays are based on the detection of platelet-activation markers, such as p-selectin (CD62p) and annexin V, exposed on the membrane of normal donor platelets upon incubation with serum/plasma samples from VITT patients. The monoclonal antibody CD62P recognizes the alfa-granule membrane protein, p-selectin, translocated to the platelet upon activation [14]. In previous studies [15,16], the functional FC assay was used to confirm the presence of functional relevant antibodies in patients with clinical features of HIT, contributing to a fast and reliable diagnosis. In other studies, FC displayed very high sensitivity and specificity values (88 and 95%, respectively) [17].

The aim of this study was to evaluate the role of FC as a functional test to confirm the diagnosis of VITT in a series of VITT patients investigated in two Italian centers.

## 2. Methods

### 2.1. Study Population

The study was performed in two Italian centers: Azienda Ospedaliera Universitaria Careggi in Florence and Fondazione Policlinico Universitario Agostino Gemelli IRCCS in Rome.

We tested 5 patients from Rome (4 M/1 F; median age 42 (33–67)) and 8 patients from Florence (2 M/6 F, median age 71 (41–78)) who developed a clinical VITT picture with both the Vaxzevria (AstraZeneca) and Ad26.COV2S (Johnson & Johnson) vaccines. The clinical and demographic characteristics of the study population are reported in Table 1.

Samples were collected before intravenous immunoglobulin (IvIg) or anticoagulant administration in 8 patients and during IvIg therapy in 5 patients.

### 2.2. Blood Collection

Whole blood (WB) from all studied subjects was collected from the antecubital vein into plastic tubes (Vacutainer) containing NaCitrate 0.109 M to obtain plasma samples and with no anticoagulant for sera samples. Samples were centrifuged at 2000× *g* for 10 min at 4 °C and then stored in aliquots at −80 °C until analysis.

### 2.3. Immunological Assays

Immunological identification of anti-PF4 antibodies was performed by using two different immunosorbent assays in use, respectively, in Careggi and in A. Gemelli Hospital, i.e., Lifecodes PF4 IgG assay (Immucor, Milan, Italy) and Asserachrom HPIA-IgG assay (Stago, Milan, Italy), according to manufacturer’s instructions. In addition, a chemiluminescence HemosIL Acustar HIT IgG assay (Werfen, Milan, Italy) was performed in all cases.

The cut-off values were <1 U/mL for HemosIL Acustar HIT IgG assay, <0.4 optical density (O.D.) for Lifecodes PF4 IgG assay. A kit-specific cut-off in relation to a kit reference plasma was used for Asserachrom HPIA-IgG assay [11].

### 2.4. Functional Assays

All samples were tested using HIPA as reported by Greinacher and co-workers [18,19] with minor modifications. Briefly, washed platelets from 5 healthy donors were incubated with patients’ VITT serum/plasma in the presence of buffer, 0.3 IU/mL heparin, 100 IU/mL heparin.

The FC assay was performed according to local protocols in use in the two institutions described below.

In the Careggi Hospital, the FC was performed as reported by Denys et al. [16] with minor modifications. Briefly, platelet-rich plasma (PRP), obtained by slow centrifugation at 250× *g* for 10 min of sodium-citrate anticoagulated WB from 4 healthy volunteers, was incubated for 40 min at room temperature in different conditions as follow: (1) PRP+ VITT serum+ PBS; (2) PRP+ VITT serum+ heparin 0.3 IU/mL; (3) PRP+ VITT serum + heparin 100 IU/mL; (4) PRP+ HIT serum+ heparin 0.3 IU/mL as positive control.

In the Gemelli Hospital, the FC assay was performed according to a previously reported method [17]. A WB sample from a healthy donor was centrifuged at 650 rpm × 15 min at room temperature to obtain PRP. Control PRP was incubated for 1 h in different conditions as follows: (1) PRP+ VITT plasma + buffer; (2) PRP+ VITT plasma + heparin 0.3 IU/mL; (3) PRP+ VITT plasma + heparin 100 IU/mL; (4) PRP+ control platelet-poor plasma (PPP)+ thrombin receptor-activating peptide (TRAP) 10 uMol/L as positive control and (5) PRP+ control PPP+ buffer as negative control.

The following protocol was used in both institutions. An aliquot of the reaction mixture was incubated with CD61 (BD biosciences) or CD42b (BD Biosciences) monoclonal antibodies for platelet identification and CD62P (BD Biosciences) monoclonal antibodies for detecting activation and analyzed by a flow cytometer (BD-FACSCanto; Cytoflex 500). Mean fluorescence intensity (MFI) was measured.

For patients recruited at the Gemelli Hospital, a modified Heparin Platelet Activation (HEPLA) index, according to a previously reported method applied to HIT patients (14), was calculated. Since heparin was not involved in the VITT cases, the index was named PLA index and was adapted to VITT and control samples as follows:

PLA index= MFI Buffer/[MFI TRAP CTRL+ − MFI PBS CTRL−](1)

MFI parameter instead of % of platelets was used, at variance with the original protocol. The PLA index may be considered a standardized expression of the results. Eighteen samples from non-VITT patients, i.e., patients investigated for thrombosis or thrombocytopenia after anti-COVID-19 vaccines with ELISA and functional assays negative for VITT, were used as controls. The mean PLA index measured with samples from 18 controls was 0.286 ± 0.124. The PLA index cut off for platelet activation, defined by the mean of control samples +2 SD of control samples, was 0.534. A patient’s plasma was considered VITT-positive if the PLA index exceeded the 0.534 value.

## 3. Results

### 3.1. Immunological Assays

All VITT patients tested negative for the AcuStar test and highly positive for anti-PF4 antibodies ELISA assays, except for patient #5*R, who was analyzed after IvIg therapy initiation (Table 2). These results confirmed that AcuStar chemiluminescence test was not able to identify VITT antibodies, as previously reported (11), whereas many commercially available ELISA tests could detect VITT antibodies (11,14).

### 3.2. Functional Assays

The HIPA testing was positive with a typical VITT pattern, i.e., non-heparin-dependent platelet activation by patient’s sample, in six patients; weakly positive in three patients, one of whom was under IvIg therapy; negative in one patient under IvIg therapy; positive in two patients with a typical HIT, i.e., heparin-dependent, platelet-activation pattern (Table 2).

**Figure 1:**
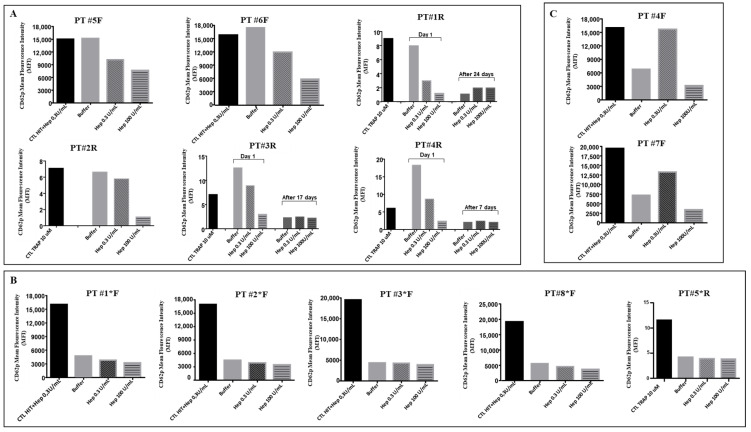
Flow cytometry platelet functional test for identification of anti-PF4/polyanions. P-selectin exposure induced by samples of 13 VITT patients. (**A**) Patients with a typical VITT pattern; (**B**) patients investigated under/after IVIG therapy; (**C**) patients with a HIT pattern. * Patients analyzed under IvIg therapy.

The FC measurement of p-selectin exposure demonstrated a typical laboratory VITT pattern, characterized by non-heparin-dependent platelet activation induced by VITT serum/plasma, in six patients. Samples from patients #5F, #6F, #1R, #2R, #3R, and #4R induced a great platelet activation in the presence of buffer only, which was reduced in the presence of therapeutic heparin dose (0.3 IU/mL) and significantly reduced in the presence of high heparin dose (100 IU/mL) (Figure 1A). Five patients who were under IvIg therapy at the time of blood withdrawal (#1*F, #2*F, #3*F, #8*F, and #5*R) had low or no platelet activation (Figure 1B). These results, which agree with previously reported studies [4,5,6,7,8,9], confirmed that the mechanism for VITT platelet activation is mediated by platelet FcγRIIa receptor binding by pathogenetic immune complex and that therapeutic IvIg effectively inhibits platelet activation by saturation of those receptor sites. In two patients with a clinical VITT picture, #4F and #7F, a typical HIT pattern was found, characterized by heparin-dependent platelet activation (Figure 1C), demonstrating that VITT antibodies cross-reacted with heparin in some cases, which is relevant for the choice of anticoagulant treatment in these patients. Interestingly, the chemiluminescent AcuStar assay was negative in all VITT patients, including those two subjects with HIT-like patterns. In three patients (#1R, #3R, and #4R), antibody testing performed several days after therapy initiation showed their absence on both ELISA and FC assays (Figure 1A).

**Figure 2:**
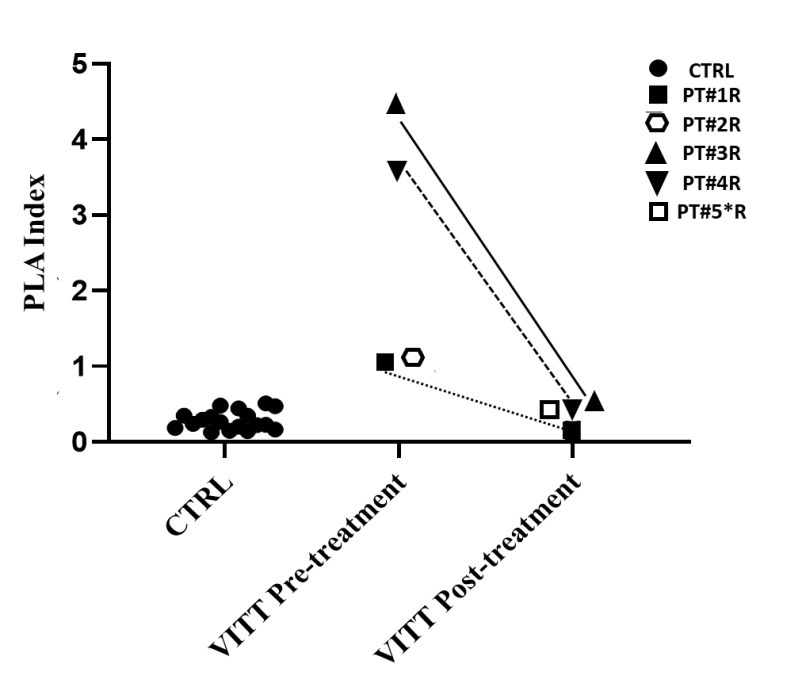
HEPLA index in 5 VITT patients compared to controls, i.e., no-VITT patients investigated for thrombosis or thrombocytopenia after Vaxzevria (AstraZeneca) and Ad26.COV2.S (Johnson & Johnson) (*n* = 18). PT#2R was investigated before death occurred after 12 h. PT#5*R was investigated 24 h after IvIg therapy had been started. * Patients analyzed under IvIg therapy.

In patients recruited at A. Gemelli Hospital, the locally used protocol allowed calculation of a PLA index [17], with the aim of standardizing results expression. In Figure 2, PLA index calculated before and several days after treatment in VITT patients is shown and compared to the index calculated in 18 control subjects, i.e., patients investigated for thrombosis or thrombocytopenia after anti-COVID-19 vaccines, whose diagnosis of VITT was not confirmed by ELISA and functional assays. Although the PLA index should be validated on a larger cohort of VITT patients, these preliminary results showed that positive and negative samples could be clearly discriminated by this index (Figure 2).

## 4. Discussion

FC seems a sensitive and reliable method to detect the ability of anti-PF4 antibodies to activate platelets. Compared to the gold standard HIPA test, results were comparable in eight cases; in three cases, FC was more sensitive than HIPA (#2R, #4R, and #2F). In one case only, HIPA was more sensitive than FC (#1F*).

A typical VITT pattern was found in most patients, with platelet activation that was non-heparin-dependent. However, in some VITT patients, antibodies cross-reacting with heparin could be documented, strongly suggesting contraindication for heparin treatment. Our results also demonstrated that the evaluation in samples from patients treated with Ig might be associated with false-negative results.

The cases reported were all with a very high pre-test probability based on: (1) type of vaccination; (2) timing from vaccination; (3) entity of thrombocytopenia; and (4) presence of thrombosis. For these reasons, it is conceivable that immunologic and functional tests were both positive, with some small differences in the entity of response at functional assays.

However, antiPF4 antibodies that do not activate platelets and that have no clinical relevance may also be documented in the general population. Hence, the simple presence of these antibodies, as determined by ELISA immunoassays, is not enough to determine that they are responsible for the disease [20]. Therefore, a definite diagnosis of VITT, similarly to HIT, is made on a positive functional test, demonstrating that the circulating antibodies detected by immunological tests are functionally able to activate platelets.

In this study, we demonstrated that FC may be used as an alternative to HIPA as a functional test for VITT. Although two different protocols with small differences were used in the two institutions, both protocols were able to reproduce the results of HIPA.

In conclusion, our study shows that the FC platelet activation assay can be reliably used for the diagnosis of VITT. Small amounts of VITT samples are required, and the test can be performed in FC equipped laboratories using PRP instead of washed platelets, making this analysis simpler and more feasible than HIPA. At last, we showed that FC allows identification of some samples containing heparin cross-reacting antibodies, thus suggesting caution for heparin treatment in those patients.

## Figures and Tables

**Table 1:** Demographic and clinical characteristics of the study population. * Patients analyzed under IvIg therapy.

VITT Cases	Age	Sex	Vaccine Type	Onset after Administration (Days)	Platelet Count	Thrombosis
Pt #1*F	57	F	AZ	6	10.000/µL	Portal vein; pulmonary embolism; splenic vein
Pt #2*F	73	F	AZ	6	10.000/µL	Pulmonary embolism; cerebral vein
Pt #3*F	75	F	AZ	7	23.000/µL	Cerebral vein; portal vein
**Pt #4F**	71	M	AZ	6	<10.000/µL	Pulmonary embolism
**Pt #5F**	78	F	JJ	10	24.000/µL	Multiple lower limb vein
**Pt #6F**	41	F	AZ	9	49.000/µL	Cerebral vein
**Pt #7F**	61	M	AZ	7	20.000/µL	Multiple lower limb vein
**Pt #8F**	59	F	AZ	9	69.000/µL	Multiple lower limb vein; pulmonary embolism
**Pt #1R**	35	F	AZ	6	20.000/µL	Right superior sinus; right transverse sinus; intrahepatic portal vessels
**Pt #2R**	67	M	AZ	11	25.000/µL	Portal vein; mesenteric veins; splenic veins
**Pt #3R**	42	M	AZ	7	33.000/µL	Superior sagittal sinus (partial); right sigmoid and transverse sinuses
**Pt #4R**	33	M	AZ	7	25.000/µL	Superior sagittal sinus; straight sinus; right transverse sinus; right jugular vein; left carotid bifurcation; pulmonary embolism; abdominal aorta
Pt #5*R	34	M	JJ	11	66.000/µL	Right sigmoid and transverse sinuses; right jugular vein

**Table 2:** Results of immunological and functional tests in VITT patients. * Patients analyzed under IvIg therapy.

VITT Case	Vaccine Type	Hemosil AcuStar HIT-IgG (n.v. ≤ 1 U/mL)	Lifecode PF4 IgG Test (O.D./Cut Off 0.4)	Asserachrom HPIA IgG ELISA Pre-Treatment (O.D./Cut Off)	Asserachrom HPIA IgG ELISA Post-Treatment (O.D./Cut Off)	Flow Cytometry (MFI)	PLA Index Pre-Treatment (Cut Off 0.534)	PLA Index Post-Treatment (Cut Off 0.534)	Hipa Test
Pt #1*F	AZ	0.04	Positive (2.472)	/	/	Weakly Positive	/	/	Positive
Pt #2*F	AZ	0.15	Positive (1.912)	/	/	Weakly Positive	/	/	Negative
Pt #3*F	AZ	0.06	Positive (3.128)	/	/	Weakly Positive	/	/	Weakly Positive
**Pt #4F**	AZ	0.04	Positive (2.942)	/	/	HIT pattern	/	/	HIT Pattern
**Pt #5F**	JJ	0.01	Positive (3.339)	/	/	Positive	/	/	Positive
**Pt #6F**	AZ	0.02	Positive (1.473)	/	/	Positive	/	/	Positive
**Pt #7F**	AZ	0.51	Positive (3.257)	/	/	HIT pattern	/	/	HIT Pattern
Pt #8*F	AZ	0.19	Positive (3.026)	/	/	Weakly Positive	/	/	Positive
**Pt #1R**	AZ	0.06	/	Positive (1.230/1.135)	Negative (0.344/0.988)	Positive	1.031	0.125	Positive
**Pt #2R**	AZ	/	/	Positive (1.330/1.135)	/	Positive	1.075	/	Weakly Positive
**Pt #3R**	AZ	0.04	/	Positive (2.723/1.135)	Negative (0.464/1.809)	Positive	4.286	0.441	Positive
**Pt #4R**	AZ	0.38	/	Positive (2.973/1.123)	Negative (0.696/1.092)	Positive	3.552	0.326	Weakly Positive
Pt #5*R	JJ	0.16	/	/	Negative (0.790/1.344)	Weakly Positive	/	0.373	/

## Data Availability

Not applicable.
